# Circulating adipokine levels and prognostic value in septic patients

**DOI:** 10.1186/s12950-016-0138-z

**Published:** 2016-09-06

**Authors:** Andreas Hillenbrand, Pengfei Xu, Shaoxia Zhou, Annette Blatz, Manfred Weiss, Sebastian Hafner, Doris Henne-Bruns, Uwe Knippschild

**Affiliations:** 1 Department of General and Visceral Surgery, University Hospital Ulm, Albert-Einstein-Allee 23, 89081 Ulm, Germany; 2 Department of Clinical Chemistry and Pathobiochemistry, University Hospital Ulm, Albert-Einstein-Allee 23, 89081 Ulm, Germany; 3 Department of Anesthesiology, University Hospital Ulm, Albert-Einstein-Allee 23, 89081 Ulm, Germany

**Keywords:** Adipokines, Adiponectin, Leptin, Sepsis

## Abstract

**Background:**

Adipokines have a wide range of effects and are linked to sepsis and septic shock. The aim of the present study was to describe the changes in adipokine levels in septic patients in relation to patients’ preseptic adipokine levels. Furthermore, we examined adipokines as prognostic markers.

**Methods:**

Fourteen consecutive critically ill patients meeting the clinical criteria for severe sepsis or septic shock 3 days up to 1 month after major visceral surgery were enrolled prospectively. Plasma adipokines were measured preoperatively, 1 and 4 days after diagnosis of severe sepsis or septic shock following elective surgery.

**Results:**

Median plasma adiponectin levels were lowered and resistin and leptin levels elevated in sepsis compared with preseptic plasma levels. MCP-1, C-reactive protein and white blood cell count were higher in septic compared with preseptic patients.

Survivors had significantly higher preseptic adipokine levels than non-survivors. Adiponectin levels of survivors decreased significant (on average by 33 %) at day one after onset of sepsis compared with preseptic levels. In contrast, median adiponectin levels of patients dying during sepsis showed a slight increase (11 %). Median BMI of survivors was 30 kg/m^2^, median BMI of non-survivors was 25, respectively.

**Conclusions:**

Adipokine levels change during the course of sepsis. Higher preseptic adiponectin levels and decreasing adiponectin levels after onset of sepsis are associated with survival of sepsis. Survival of overweight and obese patients was higher than in normal weight patients. Changes in adiponektin levels could be a prognostic marker for outcome of severe sepsis/septic shock following surgery.

## Background

Postoperative severe infection and sepsis remain a significant cause of morbidity and mortality in intensive care units. The complex dysregulated host response includes uncontrolled inflammation and immune suppression and is largely mediated by pro- and anti-inflammatory cytokines. Among others, white adipose tissue (WAT) is involved in the production of these pro- and anti-inflammatory cytokines and the contribution of WAT to the inflammatory reaction has already been demonstrated [1]. Traditionally considered as a long-term energy storing depot, WAT is now seen as an active endocrine organ that releases a large number of bioactive mediators, mainly adipokines (adipokines defined here as signaling proteinaceous factors mainly produced or released by adipose tissue) [2]. In septic patients as well as in morbidly obese patients, the expression of these adipokines is altered. Especially, the levels of pro-inflammatory adipokines are elevated [3]. In morbidly obese patients, pro-inflammatory adipokines appear to contribute strikingly to the ‘low-grade inflammatory state’, setting up a cluster of metabolic aberrations including obesity-related metabolic/cardiovascular co-morbidities that collectively define the metabolic syndrome [4]. In critically ill and septic patients, adipokines interact with inflammatory processes, and with coagulation. Furthermore, they have an impact on insulin resistance [5]. Thus, some of the metabolic abnormalities in the metabolic syndrome share several common features with those of sepsis and multiple organ dysfunction syndrome of critical illness [6].

While morbid obesity is a risk factor for many diseases, an inverse relationship between obesity and mortality has been described in patients with heart failure, coronary heart disease, diabetes, and septic shock [7, 8]. This phenomenon is known as the ‘obesity survival paradox’. A possible explanation among others could be an underlying difference in adipokine-mediated metabolic and immune response to acute illness [9]. Adipokines and their contribution to the inflammatory reaction are still under investigation.

Changes in adipokine levels in septic patients have been described often [10], but the results are somewhat contradictory. Compared with healthy controls, elevated and lowered adiponectin levels have been reported in septic patients [3, 11].

There have even been different results reported according to whether levels rise or fall [12]. In part this could be explained by the high variance of adipokine levels between individuals in the absence of normal values. Changes in adipokine levels in septic patients have been mostly reported by comparison with a control group. Therefore, the aim of the present prospective observational study was to compare changes in adipokine profile in critically ill patients with their preseptic profile.

## Methods

In this study, 14 consecutive critically ill patients meeting the clinical criteria for severe sepsis or septic shock defined by Bone [13] after major abdominal surgery procedures were prospectively enrolled between January 2011 and May 2014. All patients undergoing abdominal tumor surgery were asked to participate. 14 out of these participating 548 patients developed severe sepsis or septic shock. The Simplified Acute Physiology Score (SAPS II) was used to define the severity of disease and organ dysfunctions, respectively [14].

Blood samples were collected preoperatively in the context of a routine blood draw, and 1 day after diagnosis of severe sepsis or septic shock and every third additional day in sepsis at 7 a.m. in the fasting state following elective major visceral surgery (10 ml venous blood, collected in a chilled blood tube with Lithium Heparin). In six patients, only one septic blood sample was obtained since the underlying cause of sepsis was relieved (*n* = 3) or patients died (*n* = 3). In eight patients, a second blood sample was obtained on the fourth day of the septic course. Overall, eight out of the evaluated 14 patients died due to sepsis. The surviving six patients were discharged with no further 30-day mortality.

Plasma was obtained by centrifugation (1800 × g for 15 min), and the samples were subsequently stored in aliquots at −80 °C until further analysis. Body weight and height for calculation of body mass index (BMI) of septic patients was self-reported (or estimated if no self-report was possible). Exclusion criteria were: age < 18 years, pregnancy, rapidly progressing underlying disease such as preoperative severe liver failure, HIV/AIDS, cardiogenic shock as the primary underlying disease, underlying haematologic disease, or cytotoxic therapy given within the previous 6 months.

### Cytokine and adipocyte-derived hormone measurement and reagents

Luminex Performance Assays for leptin, resistin, adiponectin, and MCP-1 were obtained from R&D Systems GmbH, 65205 Wiesbaden, Germany.

In brief, samples were added to a mixture of color-coded beads and pre-coated with analyte-specific capture antibodies. Biotinylated detection antibodies specific to the analytes of interest were added. Thereafter, phycoerythrin-conjugated streptavidin able to bind the biothinylated detection antibodies was added. It binds to the biotinylated detection antibodies. Polystyrene beads were read on a dual-laser flow-based detection instrument (Bio-Plex® 200 system, Bio Rad Hercules, California 94547, USA) (www.rndsystems.com/product_detail_objectname_luminex_assay_principle.aspx).

### Statistical analysis

All values are expressed as median with range.

When comparing data between the three blood samples, i. e., preoperatively, septic day one and septic day four, statistical analysis was performed using the Friedman test. When comparing two blood samples or survivors with non-survivors, the Wilcoxon signed rank test was used. Analysis was performed with WinSTAT, Version 2009.1 (R. FitchSoftware) and the IBM SPSS Statistics 20.0.0 (Ehningen, Germany).

The Spearman rank-order correlation coefficient was calculated for correlation analysis. R indicates the correlation coefficient. A value of *p* < 0.05 was considered statistically significant. Correlation coefficient values between 0.3 and 0.7 (−0.3 and −0.7) indicate a moderate, and values between 0.7 and 1.0 (−0.7 and −1.0) indicate a strong, positive (negative) linear relationship.

Results are presented as box-and-whisker plots. In boxplots, the top and bottom of the rectangle represent the 25th and 75th percentile; whiskers represent the 5th and the 95th percentile, respectively. The line within the rectangle represents the median.

## Results

### Baseline characteristics of study participants

All 14 study participants underwent major surgical procedures before developing severe sepsis postoperatively, thereby meeting the criteria defined by Bone [13]. The duration between surgery and the occurrence of subsequent severe sepsis/septic shock ranged from 3 days up to 1 month (median 12 days). The patients’ baseline characteristics and diseases are shown in Table 1. Median BMI of male patients was higher compared than female BMI (29 kg/m^2^ vs. 22 kg/m^2^). The underlying causes of sepsis were anastomosis leakage (*n* = 9), pneumonia (*n* = 3), oesophagobronchial fistula after oesophageal resection (*n* = 1), and peritonitis due to an acute perforated cholecystitis (*n* = 1). The median (range) SAPS II score was of 42 (25–56) at 1 day after diagnosis of severe sepsis or septic shock.

**Table 1:** Anthropometric data of septic patients

		Age (years)	BMI (kg/m^2^)	SAPS II Score at day 1
Total (*n* = 14)	Median (min–max)	67 (51–83)	27.25 (21.5–35.5)	42 (25–56)
Female (*n* = 3)	Median (min–max)	76 (66–83)	21.5 (21.5–22.5)	37 (35–49)
Male (*n* = 11)	Median (min–max)	64 (51–79)	29.4 (24.4–35.5)	43 (25–56)
Survivors (*n* = 6)	Median (min–max)	69 (54–79)	30 (22–35)	35 (34–52)
Non-survivors (*n* = 8)	Median (min–max)	66 (51–83)	25 (22–31)	44 (25–56)

### Adipokine levels and leukocytes in the preseptic and septic state of patients

**Fig. 1:**
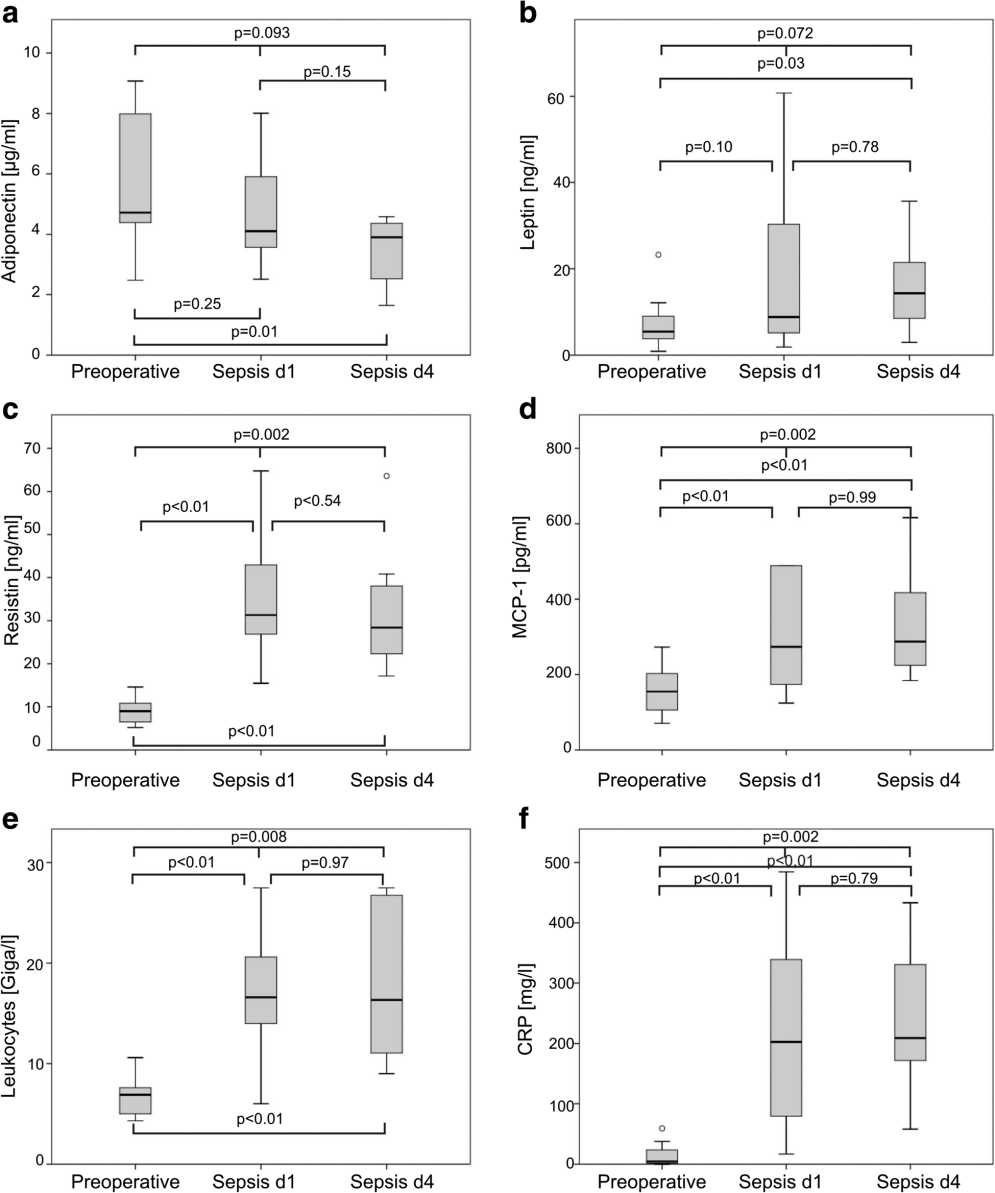
Preoperative/preseptic and postoperative/septic adipokine levels of patients (*n* = 14 for preseptic levels and at day 1 and *n* = 8 at day 4). Analysis was performed using the Friedman test when comparing the three blood samples, i. e., preoperatively, septic day one and septic day four (upper bar). When comparing two blood samples the Wilcoxon signed rank test was used. **a** Adiponectin **b** Leptin **c** Resistin **d** MCP-1 **e** Leukocytes **f** C-reactive protein

Adipokine levels were clearly altered in the septic state compared with the preseptic state. For a better overview, plasma concentrations of adiponectin, leptin, resistin, MCP-1, whole blood leukocyte count, and C-reactive protein are shown in Fig. 1. Compared with preoperative blood samples, adiponectin was lowered and resistin and leptin elevated in sepsis. Adiponectin, resistin and leptin levels did not have a strong correlation with the SAPS II score (*r* = −0.09, *p* = 0.38; *r* = 0.28, *p* = 0.17; *r* = 0.02, *p* = 0.48, respectively). BMI did not correlate significantly with adiponectin in preseptic (*r* = 0.45, *p* = 0.05) and septic states (day one: *r* = −0.28, *p* = 0.16; day four: *r* = −0.33, *p* = 0.21), but did correlate significantly with plasma leptin levels at day four of sepsis (day zero: *r* = 0.24, *p* = 0.20; day one: *r* = 0.39, *p* = 0.08; day four: *r* = 0.83, *p* = 0.01).

With regard to leptin and resistin, median levels were increased in the septic state. Resistin was elevated homogeneously in all septic patients, while leptin levels were elevated in 12 patients but decreased in two. MCP-1, C-reactive protein, and white blood cell count were significantly higher in the septic than the preseptic state. Although female levels of adiponectin and leptin have been reported to differ slightly from male levels, we found no significant differences between the two groups.

### Survival in relation to changes in adiponectin levels and BMI

**Fig. 2:**
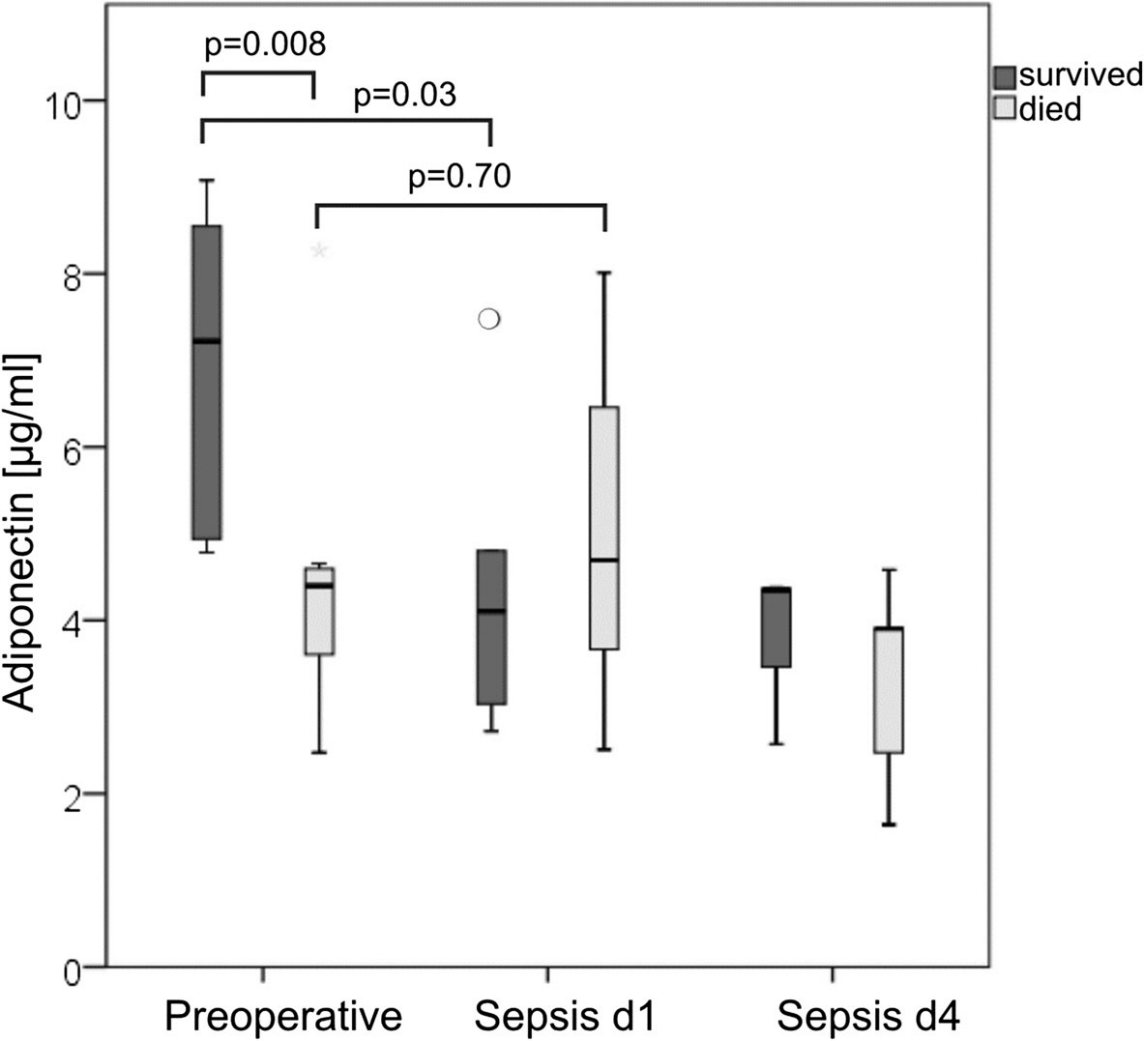
Preoperative and septic adipokine plasma levels of patients surviving sepsis (*dark*) vs. patients dying during course of sepsis (*pale*)

Although median adiponectin levels were lowered in the septic state, four patients had a considerable rise of adiponectin, and all of these four patients died of sepsis. Figure 2 compares adiponectin courses of patients surviving sepsis with patients dying in septic state. Patients who survived had significantly higher preseptic adipokine levels than patients dying in sepsis (median: 7.22 vs. 4.40 μg/ml; *p* = 0.03) and showed a significant decrease at day one (median: 4.10 μg/ml; *p* = 0.03). In patients dying during sepsis, the median adiponectin levels increased slightly at day one after onset of sepsis compared with preseptic levels (median: 4.40 vs. 4.70 μg/ml; *p* = 0.89).

Survival of overweight and obese patients was higher than in weight patients. BMI of seven patients each was ≤25 kg/m^2^ and BMI >25 kg/m^2^, respectively. In the patient group with BMI ≤25 kg/m^2^, six out of seven patients died. In the group with a BMI >25 kg/m^2^, two out of seven patients died. Overweight and obese patients had a significantly higher decrease of adiponectin after onset of sepsis than to normal weight patients (2.2 vs 0.2 μg/ml; *p* = 0.04). SAPS 2 sepsis score (without GCS) at the beginning of sever sepsis and septic shock was comparable in both BMI related groups (40 vs. 42).

## Discussion

The way that adipokines interact with inflammation in sepsis has yet to be clarified. The main results of the present study are that adiponectin levels behave inconsistently after onset of sepsis. Patients who survived sepsis had significantly higher preseptic adipokine levels than patients dying in sepsis and a significant decrease after onset of sepsis. Further, resistin and leptin levels are elevated in sepsis following major surgery.

Adiponectin exerts an anti-inflammatory effect. Several mechanisms have been suggested, including direct actions on inflammatory cells and interaction with TNF-α [5]. In septic patients, early data suggest that plasma levels of adiponectin are decreased. Whether this is a result of the disease process itself or whether patients with lower levels of this hormone are more susceptible to develop a critical illness has not yet been described [5]. Soares et al. found, that adipose cells are highly sensitive to oxidative stress, with subsequent decreased adiponectin secretion and increased lactate production, two events seen in septic patients and involved in the development of insulin resistance [15].

We found slightly lowered median plasma adiponectin levels 1 day after onset of sepsis compared with preseptic levels. In the subgroup of patients dying during the course of sepsis, we found lower preseptic adiponectin concentrations compared with patients who survived sepsis. Interestingly, in patients dying during the course of sepsis, we found slightly increased adiponectin plasma levels compared with preseptic levels at day one after onset of sepsis. Walkey et al. reported that higher systemic adiponectin concentrations on day one of critical illness are associated with lower survival in critically ill patients with respiratory failure [16]. In predicting a 28-day survival, he found adiponectin more predictive than all other factors studied, including APACHE II score. This could be explained by a dysfunctional response to the stress of early critical illness of non-survivors. In our patients, a significant initial drop in the adiponectin levels occurred during the early phase of sepsis in patients surviving sepsis (adiponectin levels of surviving patients fell by 32 %). Patients dying during the course of sepsis had an average increase of adiponectin levels of 11 % after onset of sepsis compared with preseptic levels.

Large epidemiological studies describe a correlation between BMI and adiponectin levels. In the septic state, we found no correlation of BMI with adiponectin levels. In a further septic cohort, we described similar results. Further, we reported, that adiponectin levels and insulin demand were positively correlated during sepsis [17]. Similar results have been reported by Welter et al. [18] in a prospective study of 21 septic patients. They described no differences in plasma concentrations of adiponectin in subgroups with a BMI above and below 30 kg/m^2^. Thus, the activation of the immune system may have a greater impact on plasma adiponectin levels than the obesity status.

Leptin is primarily involved in energy homeostasis [19]. However, leptin also exhibits pro-inflammatory and protective functions, and is additionally involved in the pathogenesis of a systemic inflammatory response during sepsis by several interactions towards T cells, monocytes, and cytokine production [20]. MCP-1 is expressed mainly by inflammatory cells and endothelial cells. The expression level is upregulated after pro-inflammatory stimulations. Resistin is involved in the process of inflammation by upregulating IL-6 and TNF-α and enhancing its own activity by a positive feedback mechanism [21]. Resistin relates to the severity of sepsis and the degree of inflammatory response [11]. In line with published data, we found significantly increased leptin, MCP-1, and resistin levels after onset of sepsis. However, we did not find any correlation of BMI, sepsis score, or any other analyzed parameter with leptin, MCP-1, and resistin levels.

Our study has several limitations. The study has a small sample size with a high mortality rate (57 %). Further, it is known that adipokine levels fluctuate postoperatively independently of any complication [22]. Therefore, changes in adipokine levels could not only be caused by sepsis, but also by the operative procedure itself. Furthermore, the beginning of sepsis after surgery differed between patients. The period of time in which sepsis was diagnosed was 3 days up to one month after operative procedure. This may possibly contribute to substantial bias concerning the sepsis onset levels. In addition, the majority of patients suffered from a malignant tumor, accompanied likewise by changed adipokine levels [23].

Although the results of this small study require confirmation in a larger patient cohort, we describe the variation in adipokine levels in septic patients in relation to patients’ preseptic levels. We found lowered adiponectin and elevated resistin and leptin levels in septic patients. Patients who survived sepsis had significantly higher preseptic adipokine levels than patients dying in sepsis.

## Conclusions

In severe sepsis or septic shock, adipokine plasma levels are considerably changed compared with their preseptic levels, with rising pro-inflammatory adipokine levels and decreased adiponectin levels (anti-inflammatory adipokine). The adipokine profile may differ between survivors and non-survivors of subsequent severe sepsis/septic shock following surgery.

## Abbreviations

APACHE II: Acute physiology and chronic health evaluation II
BMI: Body mass index
CRP: C-reactive protein
IL: Interleukin
MCP-1: Monocyte chemoattractant protein-1
TNF-α: Tumor necrosis factor
WAT: White adipose tissue

## References

[1] Fernandez-Riejos P, Najib S, Santos-Alvarez J, Martin-Romero C, Perez-Perez A, Gonzalez-Yanes C (2010). Role of leptin in the activation of immune cells. Mediators Inflamm.

[2] Trayhurn P, Wood IS (2004). Adipokines: inflammation and the pleiotropic role of white adipose tissue. Br J Nutr.

[3] Hillenbrand A, Knippschild U, Weiss M, Schrezenmeier H, Henne-Bruns D, Huber-Lang M (2010). Sepsis induced changes of adipokines and cytokines - septic patients compared to morbidly obese patients. BMC Surg.

[4] Fietta P, Delsante G (2013). Focus on adipokines. Theor Biol Forum.

[5] Robinson K, Prins J, Venkatesh B (2011). Clinical review: adiponectin biology and its role in inflammation and critical illness. Crit Care.

[6] Robinson K, Kruger P, Prins J, Venkatesh B (2011). The metabolic syndrome in critically ill patients. Best Pract Res Clin Endocrinol Metab.

[7] Arabi YM, Dara SI, Tamim HM, Rishu AH, Bouchama A, Khedr MK (2013). Clinical characteristics, sepsis interventions and outcomes in the obese patients with septic shock: an international multicenter cohort study. Crit Care.

[8] Nie W, Zhang Y, Jee SH, Jung KJ, Li B, Xiu Q (2014). Obesity survival paradox in pneumonia: a meta-analysis. BMC Med.

[9] Stapleton RD, Dixon AE, Parsons PE, Ware LB, Suratt BT (2010). The association between BMI and plasma cytokine levels in patients with acute lung injury. Chest.

[10] Yousef AA, Amr YM, Suliman GA (2010). The diagnostic value of serum leptin monitoring and its correlation with tumor necrosis factor-alpha in critically ill patients: a prospective observational study. Crit Care.

[11] Vassiliadi DA, Tzanela M, Kotanidou A, Orfanos SE, Nikitas N, Armaganidis A (2012). Serial changes in adiponectin and resistin in critically ill patients with sepsis: associations with sepsis phase, severity, and circulating cytokine levels. J Crit Care.

[12] Tschop J, Dattilo JR, Prakash PS, Kasten KR, Tschop MH, Caldwell CC (2010). The leptin system: a potential target for sepsis induced immune suppression. Endocr Metab Immune Disord Drug Targets.

[13] Bone RC, Balk RA, Cerra FB, Dellinger RP, Fein AM, Knaus WA (1992). Definitions for sepsis and organ failure and guidelines for the use of innovative therapies in sepsis. The ACCP/SCCM Consensus Conference Committee. American College of Chest Physicians/Society of Critical Care Medicine. Chest.

[14] Le Gall JR, Lemeshow S, Saulnier F (1993). A new Simplified Acute Physiology Score (SAPS II) based on a European/North American multicenter study. JAMA.

[15] Soares AF, Guichardant M, Cozzone D, Bernoud-Hubac N, Bouzaidi-Tiali N, Lagarde M (2005). Effects of oxidative stress on adiponectin secretion and lactate production in 3 T3-L1 adipocytes. Free Radic Biol Med.

[16] Walkey AJ, Rice TW, Konter J, Ouchi N, Shibata R, Walsh K (2010). Plasma adiponectin and mortality in critically ill subjects with acute respiratory failure. Crit Care Med.

[17] Hillenbrand A, Weiss M, Knippschild U, Stromeyer HG, Henne-Bruns D, Huber-Lang M (2011). Association of adiponectin levels and insulin demand in critically ill patients. Diabetes Metab Syndr Obes.

[18] Welters ID, Bing C, Ding C, Leuwer M, Hall AM (2014). Circulating anti-inflammatory adipokines High Molecular Weight Adiponectin and Zinc-alpha2-glycoprotein (ZAG) are inhibited in early sepsis, but increase with clinical recovery: a pilot study. BMC Anesthesiol.

[19] Farooqi IS, O’Rahilly S (2014). 20 years of leptin: human disorders of leptin action. J Endocrinol.

[20] Behnes M, Brueckmann M, Lang S, Putensen C, Saur J, Borggrefe M (2012). Alterations of leptin in the course of inflammation and severe sepsis. BMC Infect Dis.

[21] Joshi RK, Lee SA (2014). Obesity related adipokines and colorectal cancer: a review and meta-analysis. Asian Pac J Cancer Prev.

[22] Matsuda A, Matsutani T, Sasajima K, Furukawa K, Tajiri T, Tamura K (2009). Preoperative plasma adiponectin level is a risk factor for postoperative infection following colorectal cancer surgery. J Surg Res.

[23] Hillenbrand A, Fassler J, Huber N, Xu P, Henne-Bruns D, Templin M (2012). Changed adipocytokine concentrations in colorectal tumor patients and morbidly obese patients compared to healthy controls. BMC Cancer.

